# InChI – the worldwide chemical structure standard

**DOI:** 10.1186/1758-2946-6-S1-P4

**Published:** 2014-03-11

**Authors:** Stephen Heller

**Affiliations:** 1 NIST, Chemical Reference Data Group, Gaithersburg, MD 20899-83602, USA

## 

Progress of the IUPAC InChI/InChIKey project continues to move ahead with many new features and support. A collection of four short videos explaining the InChI project have been produced and are available via the InChI Trust web site. Use of the algorithm has increased over the past year to the point that numerous publications use and refer to InChI. Publicity for the project is good and has resulted in considerable increase in the usage of the InChI algorithm. The Trust web site is now available to the public and is updated regularly. Even CAS now allows for an InChI string to be used as input for a SciFinder search and the Sigma-Aldrich catalogue is InChI searchable. Extensions to the project’s current capabilities, such as InChI for chemical reactions, are being developed by a number of expert, experienced individuals and groups from various areas of chemistry. This poster presentation will describe the current technical state of the InChI algorithm and how the InChI Trust is working to assure the continued support and delivery of the InChI algorithm.

